# Co-ingestion of glutamine and leucine synergistically promotes mTORC1 activation

**DOI:** 10.1038/s41598-022-20251-2

**Published:** 2022-09-23

**Authors:** Ryoji Yoshimura, Shuichi Nomura

**Affiliations:** Department of Health and Nutrition, Faculty of Health Management, Nagasaki International University, 2825-7 Huis Ten Bosch Machi, Sasebo City, Nagasaki Japan

**Keywords:** Biochemistry, Molecular biology, Physiology

## Abstract

Leucine (Leu) regulates protein synthesis and degradation via activation of mammalian target of rapamycin complex 1 (mTORC1). Glutamine (Gln) synergistically promotes mTORC1 activation with Leu via glutaminolysis and Leu absorption via an antiporter. However, Gln has also been shown to inhibit mTORC1 activity. To resolve this paradox, we aimed to elucidate the effects of Gln on Leu-mediated mTORC1 activation. We administered Leu, Gln, tryptophan, Leu + Gln, or Leu + tryptophan to mice after 24-h fasting. The mice were then administered puromycin to evaluate protein synthesis and the gastrocnemius muscle was harvested 30 min later. Phosphorylated eukaryotic initiation factor 4E-binding protein 1, 70-kDa ribosomal protein S6 kinase 1, and Unc-51 like kinase 1 levels were the highest in the Leu + Gln group and significantly increased compared with those in the control group; however, Gln alone did not increase the levels of phosphorylated proteins. No difference in glutamate dehydrogenase activity was observed between the groups. Leu concentrations in the gastrocnemius muscle were similar in the Leu-intake groups. Our study highlights a novel mechanism underlying the promotive effect of Gln on Leu-mediated mTORC1 activation, providing insights into the pathway through which amino acids regulate muscle protein metabolism.

## Introduction

Skeletal muscle mass is integral for locomotion and is a regulator of whole-body metabolism^1^. Skeletal muscle is a locomotive organ, and decline in skeletal muscle mass is associated with decreased body movement, i.e., activities of daily living, and quality of life. Moreover, a decline in the skeletal muscle mass adversely affects metabolism because the skeletal muscles serve as a reserve of energy in the fasting state for other tissues in the form of amino acids and also increase the basal metabolic rate^2^. As these muscles are responsible for three-quarters of total glucose uptake, mass reduction decreases their blood glucose uptake, which in turn increases blood glucose levels, eventually leading to the development of diabetes^2,3^. Thus, elucidating the mechanisms underlying the regulation of skeletal muscle mass is crucial.

Generally, skeletal muscle mass increases when protein synthesis exceeds protein degradation, and dietary proteins and amino acids promote protein synthesis and suppress protein degradation via autophagy. The effect of amino acids on protein synthesis has been demonstrated in studies in rodents^4–7^, pigs^8–12^, and humans^13,14^. The effect is mediated, at least partly, by mammalian target of rapamycin complex 1 (mTORC1)^9,15,16^, which is a serine/threonine kinase and phosphorylates eukaryotic initiation factor 4E-binding protein 1 (4EBP1) and 70-kDa ribosomal protein S6 kinase 1 (S6K1). 4EBP1 binds to eukaryotic translation initiation factor 4E (eIF4E) and regulates translation initiation by inhibiting binding of eIF4E to eukaryotic translation initiation factor 4G (eIF4G), thereby preventing the formation of the active eIF4F complex, which is crucial for initiation of translation^17–19^. However, phosphorylation of 4EBP1 by mTORC1 results in the release of eIF4E from the inactive eIF4E-4EBP1 complex, thereby promoting translation. In contrast, S6K1 is activated by phosphorylation and subsequently phosphorylates ribosomal protein S6, a component of the 40S ribosomal subunit, and eukaryotic translation initiation factor 4B (eIF4B)^20^. eIF4B promotes the helicase activity of eukaryotic translation initiation factor 4A, which unwinds hairpin structures in the 5′-untranslated region.

The effect of amino acids on protein degradation is mediated by the mTORC1-regulated autophagy–lysosome pathway. Autophagy is known to be induced by amino acid starvation^21,22^. During amino acid-sufficient conditions, unc-51 like kinase 1 (ULK1) is phosphorylated and inactivated by mTORC1, which negatively regulates autophagy^23,24^. During amino acid starvation, mTORC1 dissociates from the ULK1 complex, and subsequently, autophagosome formation is initiated by the ULK1 complex^23,24^. The cytosolic microtubule-associated protein light chain 3 (LC3-I) is converted to the membrane-associated phosphatidylethanolamine-conjugated LC3 (LC3-II), which is necessary for autophagosome formation^25^.

Researchers have proposed several mechanisms underlying mTORC1 activation by the amino acids leucine (Leu)^26–30^, arginine^31^, methionine^32^, and glutamine (Gln)^27,33^, especially Leu. Among the proposed mechanisms for Leu-mediated mTORC1 activation, one is glutaminolysis via glutamate (Glu) dehydrogenase (GDH), which is activated by direct binding of Leu, which in turn promotes mTORC1 activation. Glutaminolysis involves two deamination steps: (1) glutaminase-catalyzed deamination of Gln to Glu, and (2) GDH-catalyzed conversion of Glu to 2-oxoglutarate, which activates mTORC1. In addition to GDH and 2-oxoglutarate-mediated mTORC1 activation, intracellular Gln promotes extracellular Leu absorption via bidirectional transport by the solute carrier family 7 member 5/solute carrier family 3 member 2 (SLC7A5/SLC3A2) heterodimeric bidirectional antiporter, as demonstrated by Nicklin et al.^33^; this is the rate-limiting step in mTORC1 activation. Thus, Gln may play an important role in the promotion of Leu-mediated mTORC1 activation.

In contrast, Gln synthetase was reported to inhibit mTORC1 activity and translocation of mTORC1 to the lysosomal membrane, the site of active mTORC1 localization^34^. Additionally, the same study revealed that Gln can inhibit mTORC1 activity and subsequently induce autophagy, as assessed by LC3 lipidation, i.e., LC3-II levels.

As described above, in vitro studies have reported contrasting findings about the role of Gln in mTORC1 activation. Thus, in the present study, we attempted to clarify whether co-ingestion of Gln promoted or suppressed Leu-induced mTORC1 activation in skeletal muscles. The findings of this study may help in the development of evidence-based nutritional approaches for preventing and treating muscle-related diseases, particularly sarcopenia, an aging-related disorder characterized by decreased skeletal muscle mass and function that is becoming a socially critical disease of the elderly.

## Results

### Effect of oral co-ingestion of Leu and Gln on the mTORC1 pathway and protein synthesis

**Figure 1:**
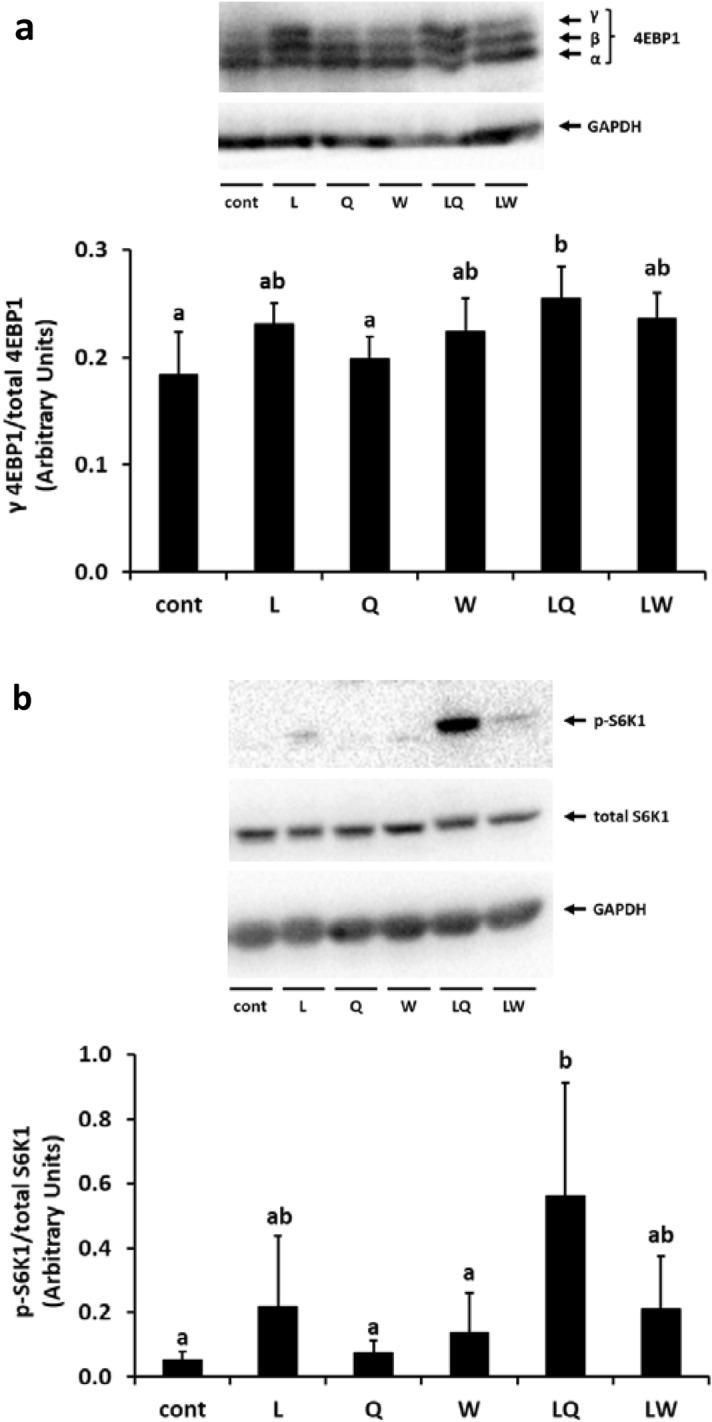
Changes in phosphorylation of 4EBP1 (**a**) and S6K1 at Thr389 (**b**) in the gastrocnemius muscle of mice administered 0.2% xanthan gum solution (Cont), leucine (L), glutamine (Q), tryptophan (W), leucine + glutamine (LQ), or leucine + tryptophan (LW) by gavage after 24 h fasting. On sodium dodecyl sulfate–polyacrylamide gel electrophoresis, 4EBP1 separated into multiple bands according to phosphorylation status. The highly phosphorylated form, γ form, exhibited the slowest mobility. Total 4EBP1 was calculated as the sum of the three bands. Densitometry results are expressed as the mean ± standard deviation (SD) (n = 5). ^a,b^Mean values denoted by different letters represent significant differences between the groups (P < 0.05). The original blots are presented in Supplementary Figs. S1 and S2.

**Figure 2:**
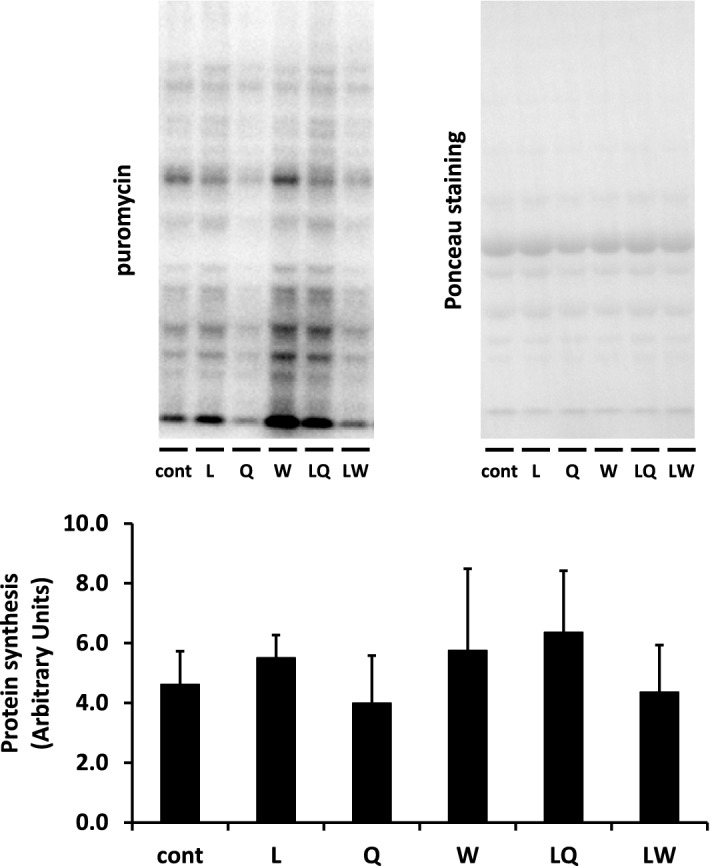
Protein synthesis evaluated by detecting puromycin-labeled peptides in the gastrocnemius muscle of mice administered 0.2% xanthan gum solution (Cont), leucine (L), glutamine (Q), tryptophan (W), leucine + glutamine (LQ), or leucine + tryptophan (LW) with gavage after 24 h fasting. Puromycin is incorporated into the nascent peptide chain; hence, quantification of puromycin incorporation was performed by measuring the density of each lane. Ponceau S staining was performed to verify equal loading of proteins. Densitometry results are expressed as the mean ± SD (n = 5). Mean values without letters are not significantly different among groups (P > 0.05). The original blots are presented in Supplementary Fig. S3.

To determine the effect of oral co-ingestion of Leu and Gln on the mTORC1 pathway and protein synthesis, we analyzed the phosphorylation of 4EBP1 and S6K1 after administration of amino acids, followed by intraperitoneal injection of puromycin, which is a protein synthesis inhibitor that is incorporated into nascent peptide chains and causes premature termination^35^. Therefore, during active protein synthesis, large amounts of puromycin-incorporated peptides can be detected by western blot analysis. The ratio of γ 4EBP1/total 4EBP1 was evaluated to analyze the effect of oral co-ingestion of Leu and Gln on the mTORC1 pathway. The γ form of 4EBP1 is the most phosphorylated form, exhibits the slowest mobility in SDS–PAGE, and cannot bind to eIF4E^36^. Moreover, bound 4EBP1 tends to dissociate from the inactive eIF4E-4EBP1 complex upon phosphorylation of 4EBP1, resulting in promotion of translation. As shown in Fig. 1a, the γ 4EBP1/total 4EBP1 ratio in the three Leu-intake groups (Leu, Leu + Gln, and Leu + Trp) was markedly higher than that in the Cont group and was significantly higher in the Leu + Gln group than in the Cont group (p < 0.05). Although Gln has been reported to increase mTORC1 activity^27,33^, no change in the γ 4EBP1/total 4EBP1 ratio was observed in the Gln group. Similarly, the level of phosphorylated S6K1 at Thr389, the mTORC1-dependent phosphorylation site^37^, also increased in the three Leu-intake groups and a marked increase was noted in the Leu + Gln group (Fig. 1b). Consistent with the above observation in the γ 4EBP1/total 4EBP1 ratio, Gln alone had no effect on S6K1 phosphorylation. Although the level of puromycin incorporation showed a trend similar to the γ 4EBP1/total 4EBP1 ratio and S6K1 phosphorylation, it did not significantly differ in any of the groups when compared to that in the Cont group (Fig. 2).

### Effect of oral co-ingestion of Leu and Gln on the autophagy–lysosome pathway

**Figure 3:**
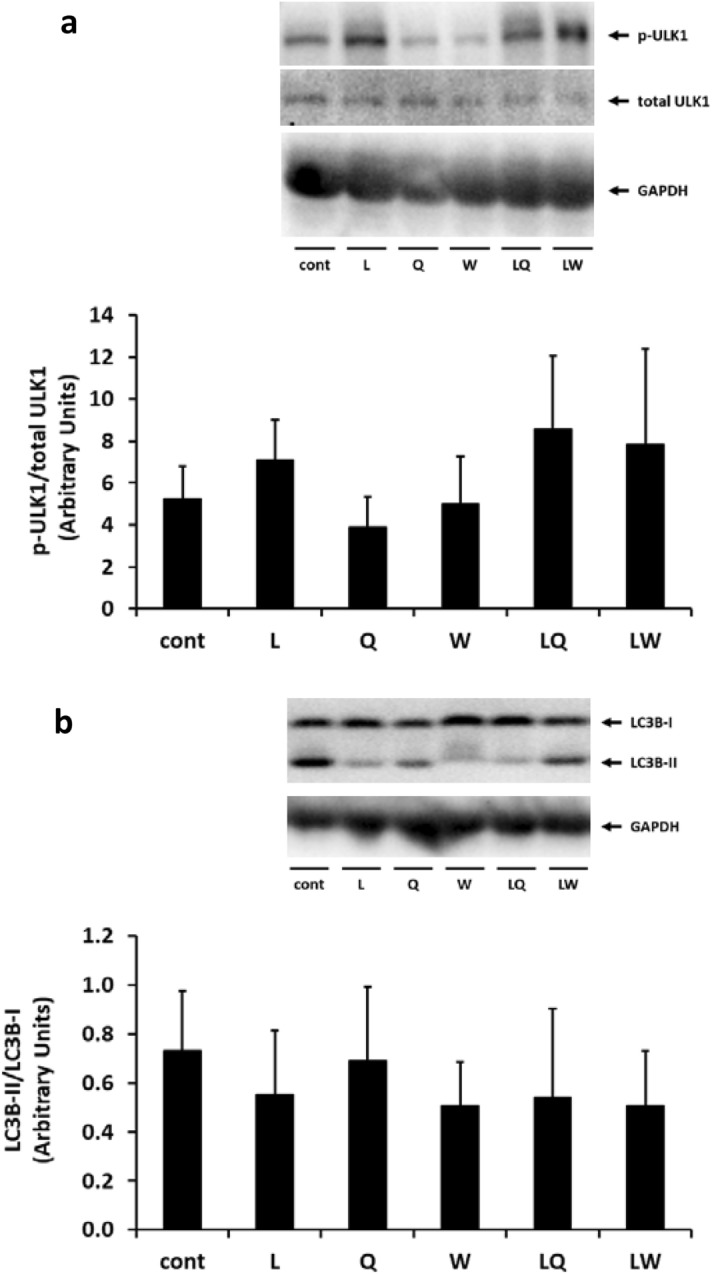
Changes in the levels of phospho-ULK1 (phosphorylated at Ser757) and LC3B in the gastrocnemius muscle of mice administered 0.2% xanthan gum solution (Cont), leucine (L), glutamine (Q), tryptophan (W), leucine + glutamine (LQ), or leucine + tryptophan (LW) by gavage after 24 h fasting. Densitometry results are expressed as the mean ± SD (n = 5). Mean values without letters are not significantly different among groups (P > 0.05). The original blots are presented in Supplementary Figs. S4 and S5.

ULK1 initiates autophagy by phosphorylating autophagy-related protein 13^25^. Leu suppresses autophagy via activation of mTORC1 and promotes the subsequent phosphorylation of ULK1 by mTORC1. In contrast, van der Vos et al.^34^ reported that Gln inhibits mTORC1 activity and activates the autophagy pathway. During autophagosome formation, LC3-I is lipidated with phosphatidylethanolamine to form LC3-II, and the conversion rate (LC3-II/I) is used as the indicator of autophagosome formation^38^. In mammals, there are at least seven orthologs of LC3, of which LC3B is well studied and widely accepted as a marker for assessment of autophagy^39^. Therefore, to clarify the effect of co-ingestion of Leu and Gln on the autophagy–lysosome pathway, we measured the levels of phosphorylated ULK1 and LC3B-II/I. As shown in Fig. 3, there were no differences in the levels of phosphorylated ULK1 and LC3B-II/I between the groups. However, levels of phosphorylated ULK1 were similar to those of 4EBP1 and S6K1 (Fig. 1), suggesting that Gln and Leu synergistically promote mTORC1 activation. Moreover, although no difference was observed among the groups, the ratio of LC3B-II/I appeared to correspond to the change in phosphorylated ULK1 levels (Fig. 3b), suggesting that ingested Gln does not promote autophagy, which is in contrast to the findings of van der Vos et al.^34^.

### Effect of amino acids on GDH activity

**Figure 4:**
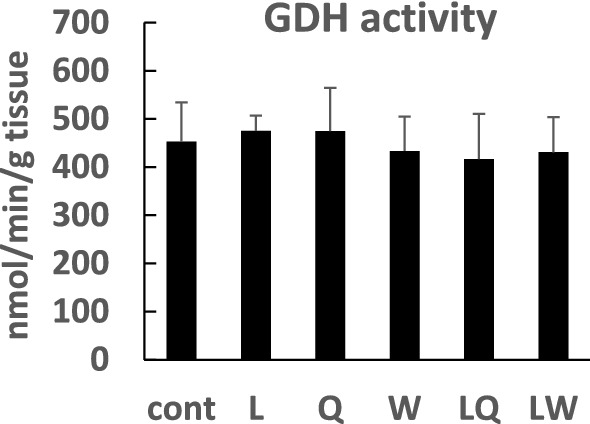
Glutamate dehydrogenase (GDH) activity in the gastrocnemius muscle of mice administered 0.2% xanthan gum solution (Cont), leucine (L), glutamine (Q), tryptophan (W), leucine + glutamine (LQ), or leucine + tryptophan (LW) by gavage after 24 h fasting. Data are expressed as the mean ± SD (n = 5). Mean values without letters are not significantly different among groups (P > 0.05).

One of the proposed mechanisms of mTORC1 activation by Leu is GDH activation via direct binding of Leu^40,41^. However, there were no significant differences in GDH activity among the groups (Fig. 4). This finding suggests that Leu-mediated mTORC1 activation observed in the present study was not related to GDH activation.

### Effect of amino acid administration on amino acid concentrations in the gastrocnemius muscle

**Figure 5:**
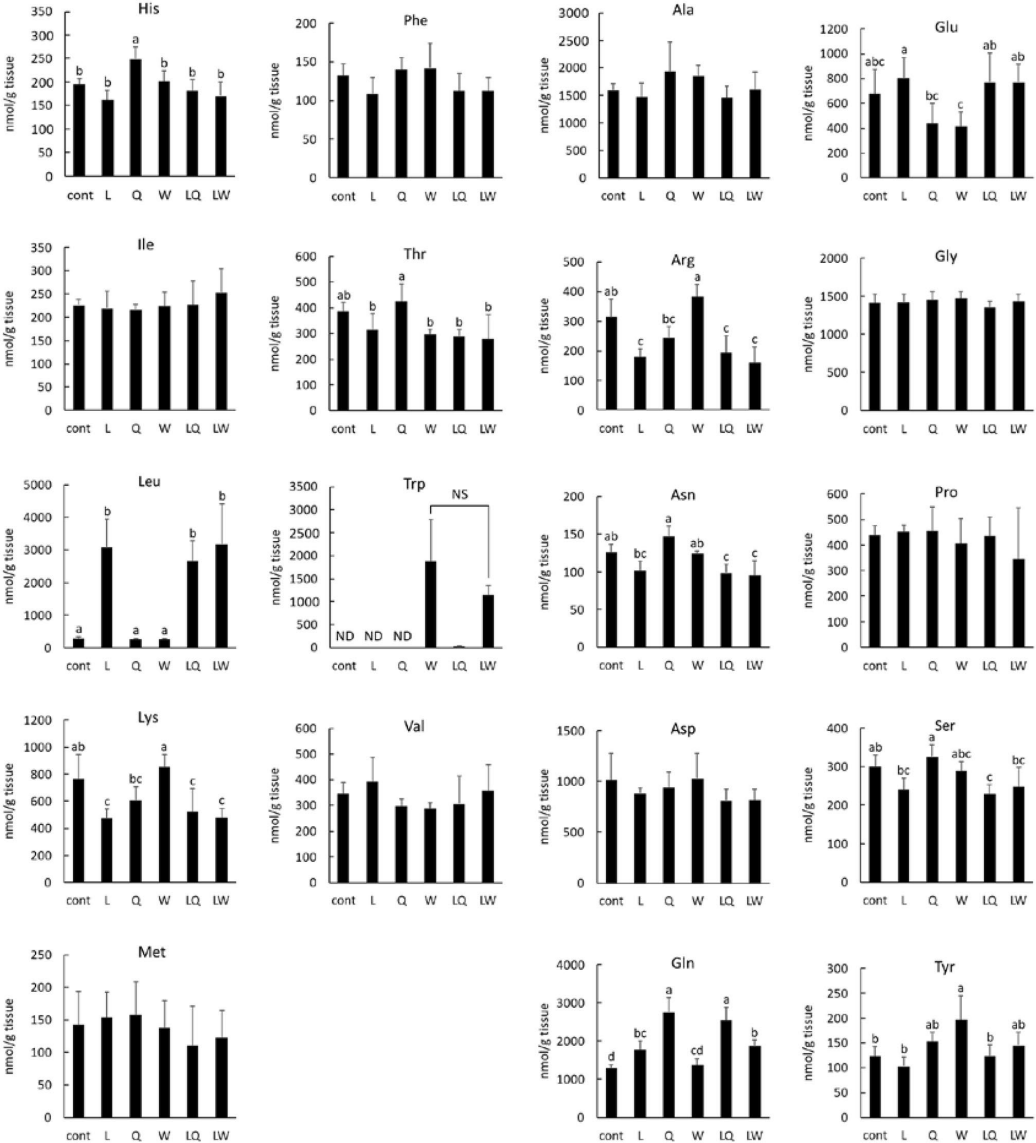
Amino acid concentrations in the gastrocnemius muscle of mice administered 0.2% xanthan gum solution (Cont), leucine (L), glutamine (Q), tryptophan (W), leucine + glutamine (LQ), or leucine + tryptophan (LW) by gavage after 24 h fasting. Data are expressed as the mean ± SD (n = 5); except for tryptophan concentration in the LQ group, where n = 1, as tryptophan was not detected in other samples. ^a,b,c,d^Mean values denoted by different letters represent significant difference between the groups (P < 0.05). Mean values without letters are not significantly different among groups (P > 0.05). *ND* not detected, *NS* not significant.

It has been reported that intracellular Gln promotes extracellular Leu absorption via a bidirectional antiporter, which is the rate-limiting step in mTORC1 activation^33^. Therefore, to clarify the effect of co-ingestion of Gln on Leu absorption, we measured amino acid concentrations in the gastrocnemius (Fig. 5). Contrary to our expectations, Leu concentration in the Leu + Gln group (2664 ± 606 nmol/g tissue) was not significantly higher than that in the Leu (3090 ± 841 nmol/g tissue) and Leu + Trp (3182 ± 1244 nmol/g tissue) groups, suggesting that the observed levels of phosphorylation of 4EBP1, S6K1, and ULK1 were not due to the effect of promotion of Leu absorption by Gln. In addition to the Leu concentration in the Leu, Leu + Gln, and Leu + Trp groups, the Gln concentration in the Gln and Leu + Gln groups, and Trp concentration in the Trp and Leu + Trp groups were elevated when compared to those in other groups in which the respective amino acid was not administered. These results indicated that the administration of Leu, Gln, and Trp increases their respective concentrations in the gastrocnemius, suggesting that the observed differences in western blotting results among groups are not due to no changes in the concentrations of the administered amino acids.

## Discussion

Leu promotes protein synthesis and suppresses protein degradation by activating mTORC1, which phosphorylates 4EBP1, S6K1, and ULK1. Some in vitro studies have reported that Gln is involved in Leu-mediated mTORC1 activation^27,33^. In contrast, it has also been reported that Gln inhibits mTORC1 in vitro by suppressing the translocation of mTORC1 to lysosomes, which is the site of localization for active mTORC1^34^. However, to the best of our knowledge, no studies had been conducted on the effect of oral intake of Gln on Leu-mediated mTORC1 activation in skeletal muscles in vivo. Therefore, in the present study, we attempted to elucidate the effect of oral intake of Gln on Leu-mediated mTORC1 activation in the skeletal muscles of mice. We observed a promotive effect of Gln on Leu-mediated upregulation of phosphorylation of 4EBP1 and S6K1, in mouse gastrocnemius muscle (Fig. 1). These results suggested that Gln promotes Leu-mediated mTORC1 activation. Additionally, it has previously been reported that Gln can activate mTORC1 via ADP ribosylation factor 1 (Arf1), which is different from the mTORC1 activation signal pathway of Leu^42^. However, in the present study, we did not observe any effect of Gln alone on mTORC1 activation, which suggests that in the conditions of the present study Arf1 did not play a critical role in mTORC1 activation. In contrast to the changes in phosphorylation levels of 4EBP1 and S6K1, the level of protein synthesis did not significantly differ in any of the groups compared with that in the control group. This similarity of protein synthesis among groups is considered to be due to the lack of provision of other amino acids comprising the body’s proteins; however, further experimental validation is needed to support this hypothesis.

In addition to 4EBP1 and S6K1, ULK1 is an mTORC1 target; however, we did not observe an increase in the levels of phosphorylated ULK1 after administration of Leu + Gln or a decrease in the levels after administration of Gln alone. The LC3B-II/ I ratio, a widely accepted marker for the assessment of autophagy, did not increase in the Leu + Gln and Gln-alone groups. Thus, these results do not support a negative regulatory role of Gln in mTORC1 activity or autophagy in the mouse skeletal muscle, which is in contrast with the results of van der Vos et al.^34^.

Next, we sought to elucidate the mechanism through which Gln promotes mTORC1 activation by Leu. It has been suggested that Leu activates mTORC1 via upregulation of deamination of Glu to alpha-ketoglutarate by the activation of GDH by direct binding of Leu to GDH^27^. However, in the present study, no difference in GDH activity was noted between the groups (Fig. 4), which suggests that GDH activity was not involved in the observed high levels of phosphorylation of 4EBP1, S6K1, and ULK1 in the Leu + Gln group. Therefore, in the present study, glutaminolysis via activation of GDH by Leu did not contribute to mTORC1 activation in the gastrocnemius muscle. On the other hand, alpha-ketoglutarate produced by GDH also plays a critical role in mTORC1 activation^27^. Thus, although there was no difference in GDH activity, the amount of alpha-ketoglutarate in the gastrocnemius muscle may have increased due to increased availability of Glu, which in turn could have affected mTORC1 activation. However, Glu concentrations in the Leu-intake groups were not significantly different (Fig. 5). Collectively, these results suggest that glutaminolysis does not contribute to the synergistic activation of mTORC1 by Leu and Gln.

It has been reported that intracellular Gln promotes extracellular Leu absorption via a bidirectional antiporter, which is the rate-limiting step that activates mTORC1^33^. However, no difference was noted in Leu concentrations among the Leu-intake groups in our study, although Gln concentrations in the Gln-intake groups increased significantly compared to those in the other groups (Fig. 5). This suggests that Gln did not promote Leu absorption via bidirectional anti-transport, even in the Leu + Gln group that showed the highest levels of phosphorylated 4EBP1, S6K1, and ULK1.

Apart from glutaminolysis and bidirectional anti-transport of Leu, other pathways including Sestrin2^30^, secretion-associated Ras-related GTPase 1B (SAR1B)^26^, leucyl-tRNA synthetase (LRS)^28^, and Src homology 3 domain-binding protein 4 (SH3BP4)^29^ have been proposed for Leu-mediated mTORC1 activation. Sestrin2 and SAR1B suppress mTORC1 activity by binding to the pentameric GATOR2 complex; however, the binding of sestrin2 and SAR1B to GATOR2 is not affected by Gln^26,30^. Similarly, SH3BP4 also acts as an inhibitor of Leu-mediated mTORC1 activation by binding to mTORC1 regulatory Ras-related GTP-binding proteins (Rags); however, it is unclear whether amino acids other than Leu affect SH3BP4^29^. On the other hand, LRS activates mTORC1 by direct binding of Leu^28^. LRS also binds isoleucine and methionine; however, the Km values for isoleucine and methionine are lower than that for Leu^43^. These findings suggest that the Km values for Gln maybe low and therefore LRS cannot bind to Gln. As shown in Figs. 1 and 3a, the Gln-alone group did not show mTORC1 activation, as assessed by lack of phosphorylation of 4EBP1, S6K1, and ULK1. Consistent with the previous results^26,30^, our results suggest that Gln does not affect the binding of sestrin2 and SAR1B to GATOR2, does not suppress SH3BP4 activity, and does not activate LRS via direct binding. Therefore, although it has been reported that Gln contributes to mTORC1 activation via glutaminolysis, extracellular Leu absorption, or Arf1, our results suggest that Gln co-ingested with Leu works synergistically with Leu to activate mTORC1 in vivo via an unknown regulatory mechanism. Further studies including omics analysis, such as phosphoproteomics analysis, using animal models are needed to elucidate the mechanism underlying the synergistic effect of Leu and Gln in mTORC1 activation and to understand the regulation of muscle protein anabolism and catabolism, because to the best of our knowledge, no study has reported on the synergistic effect of amino acids, including Gln, on Leu-mediated mTORC1 activation in vivo.

Notably, the findings of our study suggest that the previously reported Leu-mediated GDH activation-mediated mTORC1 activation is not implicated in the skeletal muscle in the conditions of the present study; however, we could not exclude the possible contribution of glutaminolysis in mTORC1 activation in skeletal muscle. Previous studies concerning the role of Leu and GDH, or glutaminolysis on mTORC1 activation were carried out with purified GDH or in cell lines^27,41,44^; hence, it is unclear whether GDH or glutaminolysis contribute to mTORC1 activation in vivo. Therefore, in addition to elucidating the mechanism underlying the synergistic effect of Leu and Gln on mTORC1 activation, further studies are needed to be clarify whether GDH or glutaminolysis contribute to mTORC1 activation in vivo. Additionally, the amount of Leu administered in the present study corresponds to the amount consumed in a 24 h period by male Sprague–Dawley rats provided free access to AIN-93 powdered diet^45^. Moreover, the same amount of Gln was also administered. Therefore, further studies are needed to verify the observed effect of Leu and Gln on mTORC1 activation using lower and physiologically realizable amounts of Leu and Gln.

The mechanisms underlying amino acid-mediated mTORC1 activation are being investigated largely in in vitro studies. Therefore, we evaluated the effect of co-ingestion of Leu and Gln on mTORC1 activation in skeletal muscle using mice and demonstrated the synergistic effect of Leu and Gln on mTORC1 activation. Furthermore, we found that the observed effect was not regulated by glutaminolysis via Leu-mediated activation of GDH or Gln-mediated promotion of Leu absorption via a bidirectional antiporter. To the best of our knowledge, we are the first to report the synergistic effect of Leu and Gln on mTORC1 activation in vivo*.* Furthermore, our results suggest that a novel mechanism underlies the observed effect.

## Methods

### Animals and experimental design

Thirty male C57BL/6J mice (seven weeks old) were purchased from Nihon S.L.C. (Hamamatsu, Japan). Mice were randomly allocated into the following groups (n = 5 per group): control (Cont), Leu-alone, Gln-alone, tryptophan (Trp)-alone, Leu + Gln, and Leu + Trp groups^46^. They were housed individually in plastic cages in an air-conditioned room at 22 ± 3 °C and 55 ± 7% humidity with a 12-h light/dark cycle (lights on from 07:00 to 19:00) under a conventional environment. Mice were acclimated for six days before use in experiments and provided ad libitum access to water and an AIN-93G composition diet (Research Diets, New Brunswick, NJ, USA ) for rodents^47^. On days 7 and 8, mice were administered saline and a 0.2% xanthan gum/0.9% NaCl solution. On day 11, after 24 h fasting^4,5,48^, mice were gastrointestinally administered 0.2% xanthan gum solution (Cont group), Leu, Gln, or Trp at 1.35 mg/10 µL of 0.2% xanthan gum solution/g body weight, or Leu + Gln or Leu + Trp at 1.35 mg + 1.35 mg/10 µL of 0.2% xanthan gum solution/g body weight by gavage. The procedure was performed as illustrated by Dey et al.^49^. Briefly, a blunt gavage needle (KN-348-20G-50, Natsume Seisakusho, Bunkyo-ku, Japan) attached to a 1-mL syringe was inserted from the oral cavity, along the palate, through to the esophagus and stomach, followed by administration of the test materials. One mouse from each group was administered in turn to mitigate the effects of sampling order; however, the experimenter could not be blinded to the treatment. After 30 min of gastrointestinal administration of amino acids, the mice were administered puromycin (Sigma-Aldrich, St. Louis, MO, USA) at 0.04 μmol/10 μL of 0.9% NaCl solution/g body weight by intraperitoneal injection^35,50,51^. Thirty minutes after intraperitoneal injection of puromycin, the mice were sacrificed by cervical dislocation and then bled by decapitation. The gastrocnemius muscle was harvested and snap-frozen in liquid nitrogen. The tissues were stored at − 80 °C until analysis.

The amount of Leu administered in these experiments was that used in previous studies on the effect of Leu on protein synthesis^4,5^ and was equivalent to the amount consumed in a 24 h period by male Sprague–Dawley rats provided free access to AIN-93 powdered diet^45^. Nitrogen is critical for amino acid biosynthesis; therefore, Trp was chosen as the amino acid to eliminate the effect of difference in nitrogen content, because, similar to Gln, it contains two nitrogen atoms. Additionally, the animals were monitored each day for normal appearance, and no unusual events were observed in any of the mice throughout the experiments.

### Western blot analysis

The gastrocnemius muscle was homogenized in 7 volumes of a buffer containing 20 mM *N*-2-hydroxyethyl piperazine-*N*′-2-ethanesulfonic acid pH 7.4, 100 mM KCl, 0.2 mM ethylenediaminetetraacetic acid, 2 mM ethylene glycol-bis (β-aminoethyl ether)-*N*,*N*,*N*9,*N*9-tetraacetic acid, 1 mM dithiothreitol, 50 mM sodium fluoride, 50 mM β-glycerophosphate, 0.1 mM phenylmethylsulphonyl fluoride, 1 mM benzamidine, 0.5 mM sodium vanadate, and 1% Nonidet P-40 using a bead homogenizer (TAITEC, Koshigaya, Japan). The insoluble material was removed by centrifugation at 10,000×*g* for 10 min at 4 °C, and the supernatant containing the extracted protein was harvested. Next, protein concentration was measured using the Bradford protein assay kit (Takara Bio, Shiga, Japan). Protein samples were mixed with 6× sample buffer (0.35 M Tris–HCl pH 6.8, 10% sodium dodecyl sulfate, 10% glycerol, 9.3% dithiothreitol, 0.012% bromophenol blue), heated for 5 min at 100 °C, and cooled on ice. Equal amounts of protein were separated by sodium dodecyl sulfate–polyacrylamide gel electrophoresis and transferred onto polyvinylidene difluoride membranes (Merck Millipore, Darmstadt, Germany). Membranes were stained with Ponceau S staining solution (0.5% Ponceau S, 1% acetic acid) to verify equal loading of proteins and then washed twice for 5 min with Tris-buffered saline (TBS) containing 0.1% Tween 20 (TBST). Membranes were blocked with blocking buffer (5% skim milk in TBST) for 1 h at room temperature (approximately 25 ± 2 °C). Subsequently, the membranes were washed three times for 5 min each with TBST and incubated with primary antibodies against 4EBP1 (#9644, 1:20,000, Cell Signaling Technology, Danvers, MA, USA), phospho-S6K1 at Thr389 (#9234, 1:1000, Cell Signaling Technology), S6K1 (#2708, 1:1000, Cell Signaling Technology), puromycin (MABE343, 1:25,000, Merck Millipore), phospho-ULK1 at Ser757 (#14202, 1:1000, Cell Signaling Technology), LC3B (#2775, 1:1000, Cell Signaling Technology), and GAPDH (#3683, 1:10,000, Cell Signaling Technology) diluted in 5% bovine serum albumin in TBST. The membranes were then washed three times for 5 min each with TBST and incubated with anti-rabbit IgG horseradish peroxidase secondary antibody (#7074, 1:2000, Cell Signaling Technology) or anti-mouse IgG horseradish peroxidase secondary antibody (#7076, 1:2000, Cell Signaling Technology) diluted in blocking buffer at room temperature (approximately 25 ± 2 °C). Next, the membranes were washed three times for 5 min each with TBST, and protein band densities were detected with an enhanced chemiluminescence reagent (Chemi-Lumi One Super, Nacalai Tesque, Kyoto, Japan) using a ChemiDoc XRC Plus system and quantified using Image Lab 5.2.1 (Bio-Rad, Hercules, CA, USA). For detection of total protein levels, after the first detection, the membranes were washed twice (5 min each time) with TBST and incubated twice (5 min each time) with stripping buffer (6 M guanidine hydrochloride, 0.2% Nonidet P-40, 10 mM dithiothreitol, and 20 mM Tris–HCl pH 7.5)^52^. The membranes were washed four times (3 min each time) with TBST, blocked, and incubated again with the appropriate primary antibody to detect the total target protein of the phosphorylated form.

### Analysis of GDH activity

A quarter of the gastrocnemius muscle (approximately 60 mg) was processed and used for measurement of GDH activity using a GDH activity assay kit (MAK099, Sigma-Aldrich), according to the manufacturer’s instructions. Absorbance was measured at 450 nm using the MULTISKAN FC microplate reader (Thermo Fisher Scientific, Waltham, MA, USA).

### Measurement of amino acid concentration

The gastrocnemius muscle was homogenized in five volumes of ice-cold 5% 5-sulfosalicylic acid using a bead homogenizer (TAITEC) and centrifuged at 10,000×*g* for 10 min at 4 °C^53^. Measurement of amino acid concentration in the supernatant was outsourced to Organization for Research Initiative and Promotion at Tottori University (Tottori, Japan) and measured using an automatic amino acid analyzer (JLC-500/V2, JEOL, Ltd., Tokyo, Japan).

### Statistical analysis

Data are expressed as the mean ± standard deviation. One-way analysis of variance and Tukey–Kramer tests for multiple comparisons were performed to determine the significance of differences. Differences were considered statistically significant at P < 0.05. All analysis was performed using the statistical software Statcel4 (OMS, Tokyo, Japan).

### Approval for animal experiments

All experimental protocols involving animals were approved by the Animal Experiment Ethics Committee of Nagasaki International University (No. 18A03). All experimental animals were handled according to institutional guidelines for the care and use of laboratory animals. This manuscript was prepared according to the Animal Research: Reporting of In Vivo Experiments guidelines (https://arriveguidelines.org).

## Supplementary Information

Supplementary Figures.

## Data Availability

The datasets generated during and/or analyzed during the current study are available from the corresponding author on reasonable request.

## References

[1] Izumiya Y (2008). Fast/Glycolytic muscle fiber growth reduces fat mass and improves metabolic parameters in obese mice. Cell Metab..

[2] Kamei Y, Hatazawa Y, Uchitomi R, Yoshimura R, Miura S (2020). Regulation of skeletal muscle function by amino acids. Nutrients.

[3] DeFronzo RA (1988). Lilly lecture 1987. The triumvirate: Beta-cell, muscle, liver. A collusion responsible for NIDDM. Diabetes.

[4] Anthony JC (2002). Orally administered leucine enhances protein synthesis in skeletal muscle of diabetic rats in the absence of increases in 4E-BP1 or S6K1 phosphorylation. Diabetes.

[5] Yoshimura R (2016). Phosphorylation of 4EBP by oral leucine administration was suppressed in the skeletal muscle of PGC-1α knockout mice. Biosci. Biotechnol. Biochem..

[6] Crozier SJ, Kimball SR, Emmert SW, Anthony JC, Jefferson LS (2005). Oral leucine administration stimulates protein synthesis in rat skeletal muscle. J. Nutr..

[7] Yoshizawa F, Kimball SR, Vary TC, Jefferson LS (1998). Effect of dietary protein on translation initiation in rat skeletal muscle and liver. Am. J. Physiol..

[8] Escobar J (2005). Physiological rise in plasma leucine stimulates muscle protein synthesis in neonatal pigs by enhancing translation initiation factor activation. Am. J. Physiol. Endocrinol. Metab..

[9] Suryawan A (2008). Leucine stimulates protein synthesis in skeletal muscle of neonatal pigs by enhancing mTORC1 activation. Am. J. Physiol. Endocrinol. Metab..

[10] Murgas Torrazza R (2010). Leucine supplementation of a low-protein meal increases skeletal muscle and visceral tissue protein synthesis in neonatal pigs by stimulating mTOR-dependent translation initiation. J. Nutr..

[11] Yin Y (2010). Supplementing L-leucine to a low-protein diet increases tissue protein synthesis in weanling pigs. Amino Acids.

[12] Escobar J, Frank JW, Suryawan A, Nguyen HV, Davis TA (2007). Amino acid availability and age affect the leucine stimulation of protein synthesis and eIF4F formation in muscle. Am. J. Physiol. Endocrinol. Metab..

[13] Rieu I (2006). Leucine supplementation improves muscle protein synthesis in elderly men independently of hyperaminoacidaemia. J. Physiol..

[14] Koopman R (2005). Combined ingestion of protein and free leucine with carbohydrate increases postexercise muscle protein synthesis in vivo in male subjects. Am. J. Physiol. Endocrinol. Metab..

[15] Kimball SR (2000). Feeding stimulates protein synthesis in muscle and liver of neonatal pigs through an mTOR-dependent process. Am. J. Physiol. Endocrinol. Metab..

[16] Ham DJ, Caldow MK, Lynch GS, Koopman R (2014). Arginine protects muscle cells from wasting in vitro in an mTORC1-dependent and NO-independent manner. Amino Acids.

[17] Sonenberg N, Hinnebusch AG (2009). Regulation of translation initiation in eukaryotes: Mechanisms and biological targets. Cell.

[18] Roux PP, Topisirovic I (2012). Regulation of mRNA translation by signaling pathways. Cold Spring Harb. Perspect. Biol..

[19] Chen J (2016). The molecular choreography of protein synthesis: Translational control, regulation, and pathways. Q. Rev. Biophys..

[20] Magnuson B, Ekim B, Fingar DC (2012). Regulation and function of ribosomal protein S6 kinase (S6K) within mTOR signalling networks. Biochem. J..

[21] Martinet W, De Meyer GR, Herman AG, Kockx MM (2005). Amino acid deprivation induces both apoptosis and autophagy in murine C2C12 muscle cells. Biotechnol. Lett..

[22] Munafó DB, Colombo MI (2001). A novel assay to study autophagy: Regulation of autophagosome vacuole size by amino acid deprivation. J. Cell Sci..

[23] Dikic I, Elazar Z (2018). Mechanism and medical implications of mammalian autophagy. Nat. Rev. Mol. Cell Biol..

[24] Lee DE, Bareja A, Bartlett DB, White JP (2019). Autophagy as a therapeutic target to enhance aged muscle regeneration. Cells.

[25] Meijer AJ, Lorin S, Blommaart EF, Codogno P (2015). Regulation of autophagy by amino acids and MTOR-dependent signal transduction. Amino Acids.

[26] Chen J (2021). SAR1B senses leucine levels to regulate mTORC1 signalling. Nature.

[27] Durán RV (2012). Glutaminolysis activates Rag-mTORC1 signaling. Mol. Cell.

[28] Han JM (2012). Leucyl-tRNA synthetase is an intracellular leucine sensor for the mTORC1-signaling pathway. Cell.

[29] Kim YM (2012). SH3BP4 is a negative regulator of amino acid-Rag GTPase-mTORC1 signaling. Mol. Cell.

[30] Wolfson RL (2016). Sestrin2 is a leucine sensor for the mTORC1 pathway. Science.

[31] Chantranupong L (2016). The CASTOR proteins are arginine sensors for the mTORC1 pathway. Cell.

[32] Gu X (2017). SAMTOR is an S-adenosylmethionine sensor for the mTORC1 pathway. Science.

[33] Nicklin P (2009). Bidirectional transport of amino acids regulates mTOR and autophagy. Cell.

[34] van der Vos KE (2012). Modulation of glutamine metabolism by the PI(3)K-PKB-FOXO network regulates autophagy. Nat. Cell Biol..

[35] Goodman CA (2011). Novel insights into the regulation of skeletal muscle protein synthesis as revealed by a new nonradioactive in vivo technique. FASEB J..

[36] Lin TA (1994). PHAS-I as a link between mitogen-activated protein kinase and translation initiation. Science.

[37] Saitoh M (2002). Regulation of an activated S6 kinase 1 variant reveals a novel mammalian target of rapamycin phosphorylation site. J. Biol. Chem..

[38] Klionsky DJ (2012). Guidelines for the use and interpretation of assays for monitoring autophagy. Autophagy.

[39] Schaaf MB, Keulers TG, Vooijs MA, Rouschop KM (2016). LC3/GABARAP family proteins: Autophagy-(un)related functions. FASEB J..

[40] Tomita T, Kuzuyama T, Nishiyama M (2011). Structural basis for leucine-induced allosteric activation of glutamate dehydrogenase. J. Biol. Chem..

[41] Prough RA, Culver JM, Fisher HF (1973). The mechanism of activation of glutamate dehydrogenase-catalyzed reactions by two different, cooperatively bound activators. J. Biol. Chem..

[42] Jewell JL (2015). Metabolism. Differential regulation of mTORC1 by leucine and glutamine. Science.

[43] Chen X (2011). Modular pathways for editing non-cognate amino acids by human cytoplasmic leucyl-tRNA synthetase. Nucleic Acids Res..

[44] Lorin S (2013). Glutamate dehydrogenase contributes to leucine sensing in the regulation of autophagy. Autophagy.

[45] Anthony JC, Anthony TG, Layman DK (1999). Leucine supplementation enhances skeletal muscle recovery in rats following exercise. J. Nutr..

[46] Ishikawa T (2017). Muscle-specific deletion of BDK amplifies loss of myofibrillar protein during protein undernutrition. Sci. Rep..

[47] Reeves PG, Nielsen FH, Fahey GC (1993). AIN-93 purified diets for laboratory rodents: Final report of the American Institute of Nutrition ad hoc writing committee on the reformulation of the AIN-76A rodent diet. J. Nutr..

[48] Sato T, Ito Y, Nagasawa T (2013). Regulation of skeletal muscle protein degradation and synthesis by oral administration of lysine in rats. J. Nutr. Sci. Vitaminol. (Tokyo).

[49] Dey TK, Karmakar BC, Sarkar A, Paul S, Mukhopadhyay AK (2021). A mouse model of *Helicobacter pylori* infection. Methods Mol. Biol..

[50] Hulmi JJ (2013). Muscle protein synthesis, mTORC1/MAPK/Hippo signaling, and capillary density are altered by blocking of myostatin and activins. Am. J. Physiol. Endocrinol. Metab..

[51] Hayasaka M, Tsunekawa H, Yoshinaga M, Murakami T (2014). Endurance exercise induces REDD1 expression and transiently decreases mTORC1 signaling in rat skeletal muscle. Physiol. Rep..

[52] Yeung YG, Stanley ER (2009). A solution for stripping antibodies from polyvinylidene fluoride immunoblots for multiple reprobing. Anal. Biochem..

[53] Naito T, Kuma A, Mizushima N (2013). Differential contribution of insulin and amino acids to the mTORC1-autophagy pathway in the liver and muscle. J. Biol. Chem..

