# Supplementary material for: Co-ingestion of glutamine and leucine synergistically promotes mTORC1 activation
Source: Sci Rep. 2022 Sep 23;12:15870. doi: 10.1038/s41598-022-20251-2 (PMC9508252; doi:10.1038/s41598-022-20251-2)
Supplement: Supplementary file 1 — Supplementary Figures. [file 41598_2022_20251_MOESM1_ESM.pdf]

Co -ingestion of glutamine and leucine synergistically promotes mTORC1 activation

Ryoji Yoshimura, Shuichi Nomura

Department of Health and Nutrition, Faculty of Health Management, Nagasaki International University, 2825-7 Huis Ten Bosch Machi, Sasebo City, Nagasaki, Japan

Address correspondence to Ryoji Yoshimura, yoshimurar@niu.ac.jp

Supplementary Figure S1. Full-length blots of Figure 1 a.

4EBP1

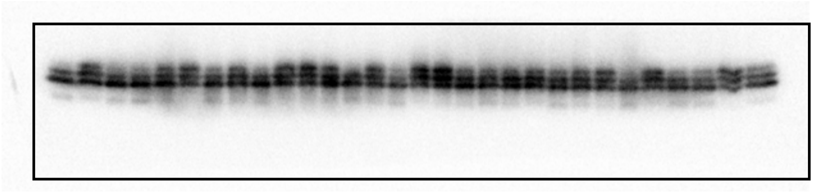

Short exposure

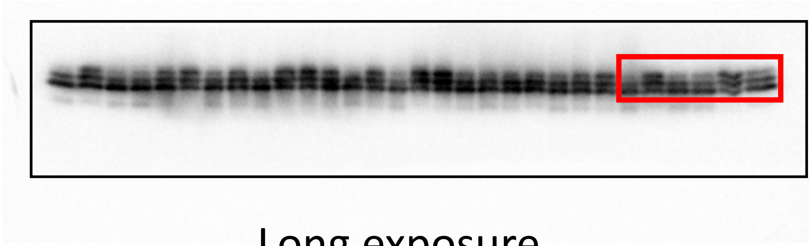

Long exposure

GAPDH

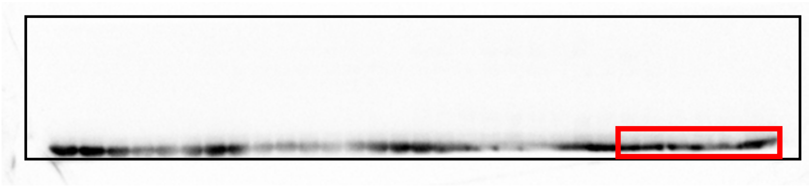

Short exposure

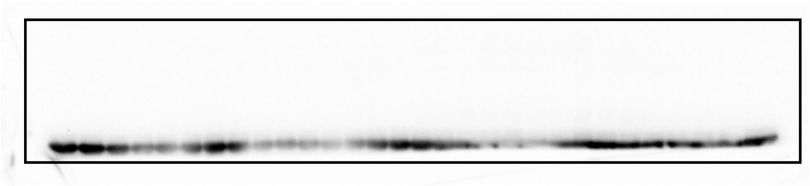

Long exposure

The red circle indicates the image inserted in the article.

Supplementary Figure S2. Full-length blots of Figure 1 b.

p-S6K1

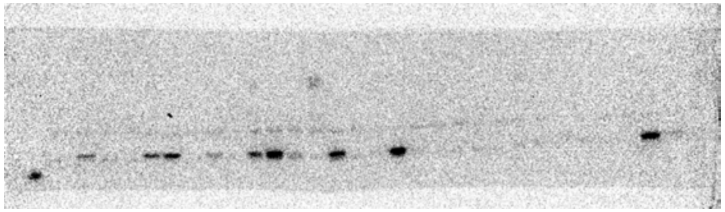

Short exposure

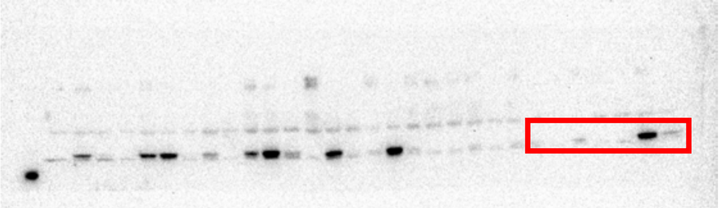

Long exposure

total S6K1

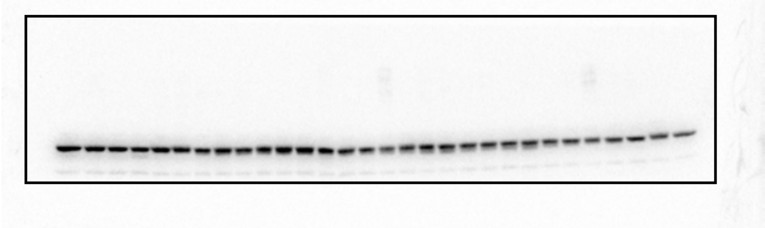

Short exposure

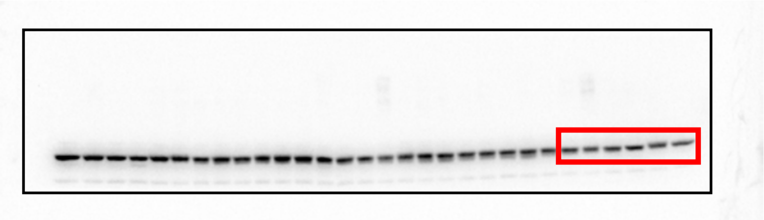

Long exposure

GAPDH

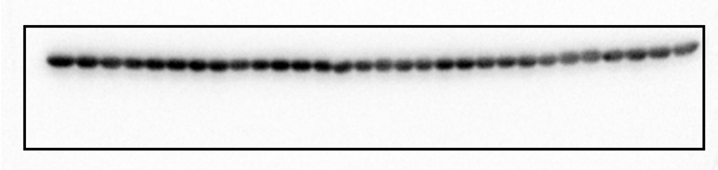

Short exposure

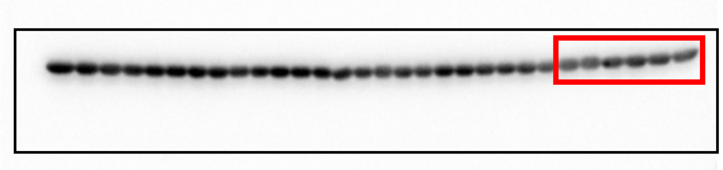

Long exposure

The red circle indicates the image inserted in the article.

Supplementary Figure S3. Full-length blots of Figure 2.

puromycin

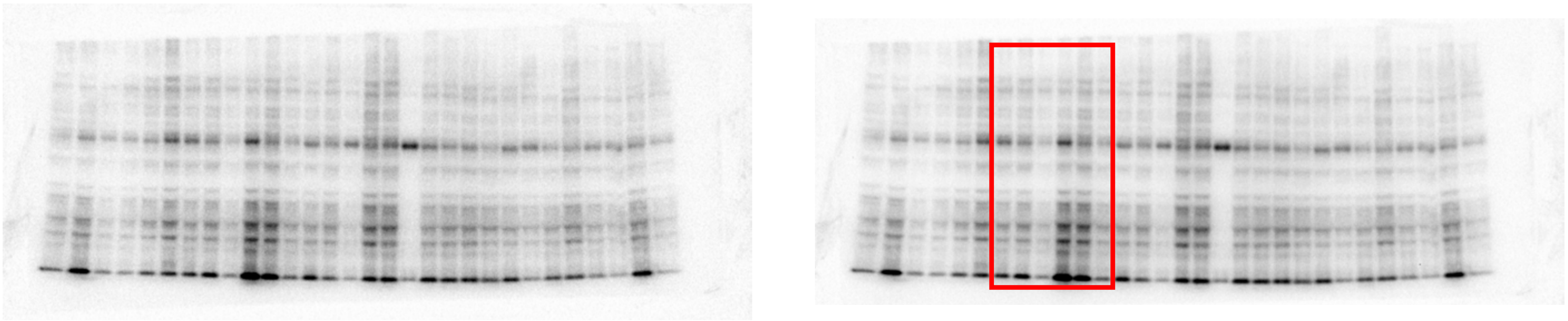

Short exposure

Long exposure

Ponceau staining

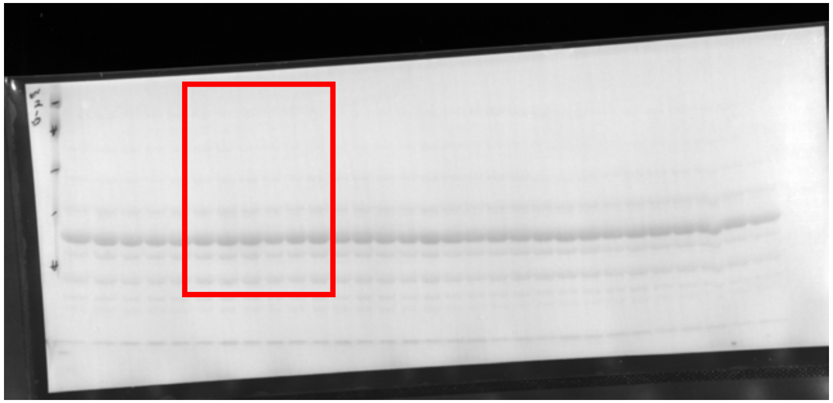

The red circle indicates the image inserted in the article. Because ponceau staining image is captured in a bright field and does not need ECL reagent, a short or long exposure image does not exist.

Supplementary Figure S4. Full-length blots of Figure 3 a.

p-ULK1

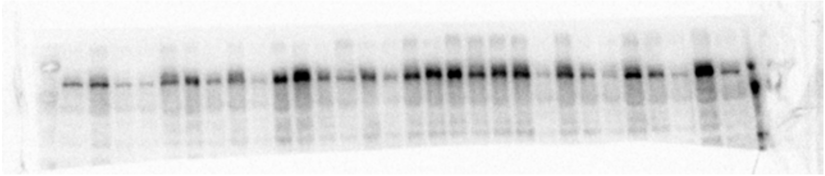

Short exposure

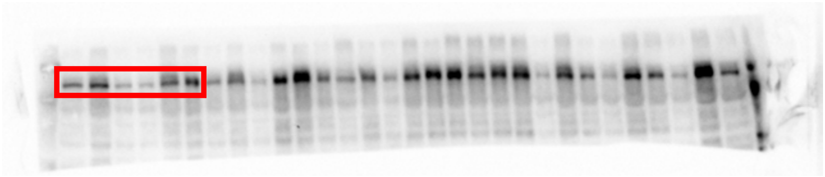

Long exposure

total ULK1

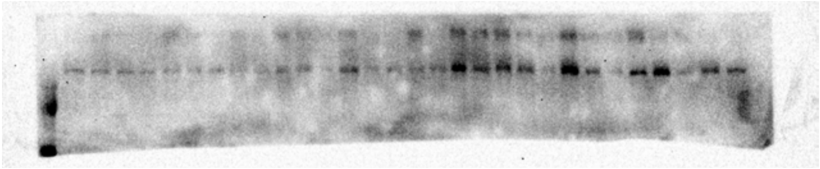

Short exposure

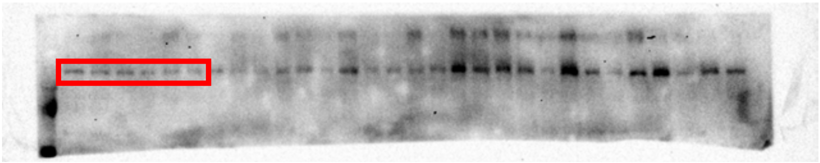

Long exposure

GAPDH

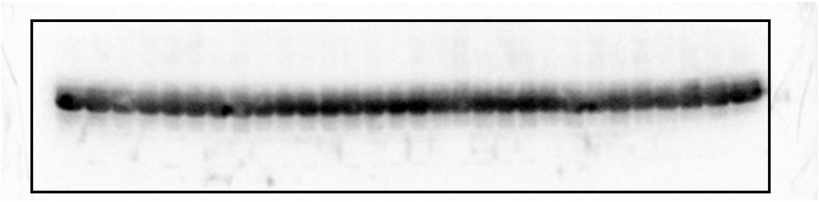

Short exposure

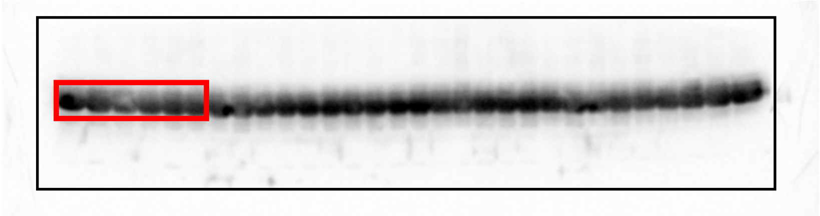

Long exposure

The red circle indicates the image inserted in the article.

Supplementary Figure S5. Full-length blots of Figure 3 b.

LC3B

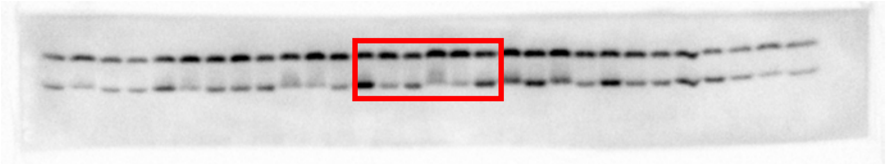

Short exposure

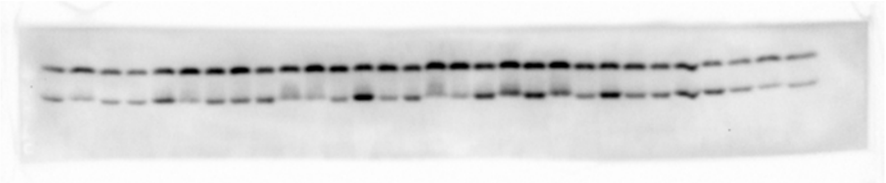

Long exposure

GAPDH

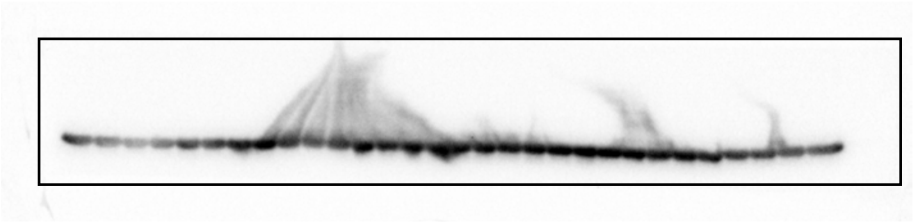

Short exposure

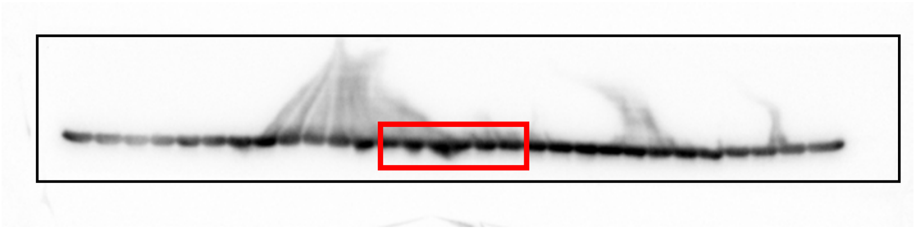

Long exposure

The red circle indicates the image inserted in the article.
